# Charles Edward Kynaston Herapath

**Published:** 1946

**Authors:** 


### Obituary

### CHARLES EDWARD KYNASTON HERAPATH, M.C., M.D. (Lond.)

Charles Herapath came of a distinguished Bristol family of scientists and doctors. William Herapath held the chair of Forensic Medicine and Toxicology (jointly with Dr. J. A. Symonds) in the Bristol Medical School. He died in 1868. His son, William Bird Herapath, was famous for his researches on quinine salts and became F.R.S. W.B.'s son, Dr. C. K. C. Herapath, made a name for himself as a general practitioner in Bristol and was the father of the subject of this memoir.

Born in 1882, he was educated at Clifton College. He left school at the age of sixteen, when he had passed the London Matriculation, and was educated medically at the University College, Bristol, and the B.R.I. He qualified in 1907, and after House appointments at the B.R.I. (he was a brilliant House Physician) he went to London in 1910 and passed the M.D. examination in the University of London. When he came back, he joined the staff of the Bristol Dispensary, but kept in close touch with the B.R.I. He was a very welcome visitor to the residents of those days and it enabled him to keep in contact with Cardiology.

At the outbreak of the first world war he, in company with his friends Reggie Statham, John Harty, Henry Goodden and others, left the B.R.I. for the services. He did not leave his company in the Third South Midland Field Ambulance throughout the whole war. He was awarded the M.C. and gained the highest esteem and affection from his men.

Soon after his return from the war, he gave up his General Practice and Dispensary appointment and started his real career as Consulting Physician and Cardiologist. He was appointed Assistant Physician, and later Physician, to the B.R.I. He held many posts in outlying hospitals, and was the Dean of the Faculty at the B.R.I. from 1934 onwards. He was secretary to the Bath & Bristol branch of the B.M.A. from 1923–34, and was also a member of the Bristol Medico-Chirurgical Society, the Association of Physicians, and the Cardiological Society. A most zealous member of the latter, he was held in high esteem by his fellow Cardiologists. He was an enthusiastic Mason and joined St. Vincent Lodge, in due course passing through the Chair and attaining high rank in the Province of Bristol.

Charles Herapath's honesty and complete incapacity for any intrigue was patent to all. His unbending integrity earned him the respect and esteem of all his colleagues. In his practice he was well-liked by his patients, and few people knew of his numerous acts of kindness to those in straitened circumstances.

He had always a love of "gadgets," and it was MacKenzie's Polygraph, just introduced when he was a House Physician, which led him into Cardiology. How well the writer remembers him coaxing that rather temperamental instrument into activity and the joy with which he examined his tracings! Later on, he naturally took to the Electrocardiograph and mastered the vagaries of the string Galvanometer.

An early possessor of a motor-bicycle, his chief joy was tuning it up ; and he was extremely scornful of the writer, who rode a similar machine but did not understand how or why it went. He built himself a wireless set in the early days—a fearsome contraption of wires and valves, which nevertheless worked satisfactorily.

He was also very fond of ceremonial, and it was this feature that really intrigued him as a territorial officer ; but he made himself highly efficient in all other activities connected with his duties. It was a great disappointment to him that, after the first world war, the Third South Midland Field Ambulance was disbanded by the Geddes axe. He would have enjoyed commanding it and would have done it very well. His Masonic activities were undoubtedly another symptom of his love of ceremonial, and he even enjoyed the University functions in academic dress.

Very happy in his home and marriage (he married Miss Phyllis Bracey of Great Yarmouth in 1921), he intended to retire into the country and acquired a property at Keynsham. Soon after the outbreak of the second world war he was obviously a sick man, but his courage and pertinacity enabled him to do his work as well as ever. It was a tragedy that he never lived to enjoy his garden in the country.

He leaves a widow, a daughter and a son ; the latter is a fourth year medical student at Bristol University. We offer to them our deepest sympathy.

